# MCL-1 is required throughout B-cell development and its loss sensitizes specific B-cell subsets to inhibition of BCL-2 or BCL-XL

**DOI:** 10.1038/cddis.2016.237

**Published:** 2016-08-25

**Authors:** Ingela B Vikström, Anne Slomp, Emma M Carrington, Laura M Moesbergen, Catherine Chang, Gemma L Kelly, Stefan P Glaser, J H Marco Jansen, Jeanette H W Leusen, Andreas Strasser, David C S Huang, Andrew M Lew, Victor Peperzak, David M Tarlinton

**Affiliations:** 1 The Walter and Eliza Hall Institute of Medical Research, Parkville, VIC, Australia; 2 Department of Medical Biology, University of Melbourne, Parkville, VIC, Australia; 3 Laboratory of Translational Immunology, University Medical Center, Utrecht, The Netherlands

## Abstract

Pro-survival BCL-2 family members protect cells from programmed cell death that can be induced by multiple internal or external cues. Within the haematopoietic lineages, the BCL-2 family members BCL-2, BCL-XL and MCL-1 are known to support cell survival but the individual and overlapping roles of these pro-survival BCL-2 proteins for the persistence of individual leukocyte subsets *in vivo* has not yet been determined. By combining inducible knockout mouse models with the BH3-mimetic compound ABT-737, which inhibits BCL-2, BCL-XL and BCL-W, we found that dependency on MCL-1, BCL-XL or BCL-2 expression changes during B-cell development. We show that BCL-XL expression promotes survival of immature B cells, expression of BCL-2 is important for survival of mature B cells and long-lived plasma cells (PC), and expression of MCL-1 is important for survival throughout B-cell development. These data were confirmed with novel highly specific BH3-mimetic compounds that target either BCL-2, BCL-XL or MCL-1. In addition, we observed that combined inhibition of these pro-survival proteins acts in concert to delete specific B-cell subsets. Reduced expression of MCL-1 further sensitized immature as well as transitional B cells and splenic PC to loss of BCL-XL expression. More markedly, loss of MCL-1 greatly sensitizes PC populations to BCL-2 inhibition using ABT-737, even though the total wild-type PC pool in the spleen is not significantly affected by this drug and the bone marrow (BM) PC population only slightly. Combined loss or inhibition of MCL-1 and BCL-2 reduced the numbers of established PC >100-fold within days. Our data suggest that combination treatment targeting these pro-survival proteins could be advantageous for treatment of antibody-mediated autoimmune diseases and B-cell malignancies.

Studies in cell lines and primary cells have revealed that when overexpressed, all pro-survival BCL-2 family members, BCL-2, BCL-XL, BCL-W, A1 and MCL-1, are capable of inhibiting the mitochondrial apoptotic pathway.^1^ Transgenic expression (using the immunoglobulin heavy chain gene enhancer, *Eμ*, or the *Vav* gene promoter, *VavP*) of BCL-2, BCL-XL or MCL-1 results in increased abundance of immature, transitional and mature B-cell subsets,^2, 3, 4^ although the marginal zone B cells were not affected by excess BCL-2 or BCL-XL.^5^ A detailed analysis of the individual and overlapping contributions of pro-survival BCL-2 family members to normal lymphocyte persistence *in vivo*, however, has been hampered by their necessity during embryonic development. Complete loss of MCL-1 in mice results in embryonic lethality prior to day 3.5,^6^ whereas BCL-XL-deficient mice die around embryonic day 14.^7^ BCL-2-deficient mice, although born at the expected frequency, succumb to polycystic kidney disease, which commences during embryogenesis.^8, 9^ The relevance of BCL-W expression to lymphocyte differentiation appears limited, as lymphoid development in BCL-W-deficient mice is normal.^10^ This is probably due to the low expression of BCL-W in normal as well as malignant lymphoid cells.^11^ The generation of A1-deficient mice has proven difficult owing to quadruplication at the gene locus in mice, but transgenic RNA interference-mediated knockdown of A1 in mice revealed a role for A1 in the maintenance of mature follicular B cells.^12^ The recent use of the BH3-mimetic compounds (ABT-263/Navitoclax: inhibits BCL-2, BCL-XL and BCL-W and ABT-199/Venetoclax: inhibits BCL-2) to treat B-cell malignancies, such as chronic lymphocytic leukemia (CLL) and non-Hodgkin lymphoma,^13, 14^ and programs to develop MCL-1-specific BH3-mimetics, suggest that the detailed analysis of the role of individual BCL-2 family proteins in the maintenance of healthy B cells should be useful in aligning the use of such drugs with particular disorders and in predicting damage to normal B-cell subsets imposed by such therapies.

We have previously shown that, with the appropriate genetic configuration, the *Mcl1* gene can be efficiently deleted in the B-cell lineage *in vivo.*^15, 16^ In the current study we used this strategy to delete the *Mcl1* and/or *Bclx* genes in an inducible fashion, with and without concomitant treatment with the BH3-mimetic compound ABT-737, to assess the individual and overlapping roles of these pro-survival BCL-2 family members in the maintenance of different B-cell subsets. From these experiments, we have learnt that BCL-XL is of only limited importance in B-cell development, that MCL-1 is required at multiple stages and that BCL-2, the main target of ABT-737 in mice *in vivo*,^17, 18^ promotes survival of some terminally differentiated Blimp-1^hi^ PC, in addition to promoting survival of mature follicular and recirculating B cells. Moreover, we found that deletion of one allele of *Mcl1* sensitizes transitional and mature B cells to loss of BCL-XL. Furthermore, deletion of both alleles of *Mcl1* greatly sensitized established plasma cells (PC) to treatment with ABT-737, even though the total PC pool in wild-type mice was not significantly affected by exposure to this drug.^19^

## Results

**Figure 1:**
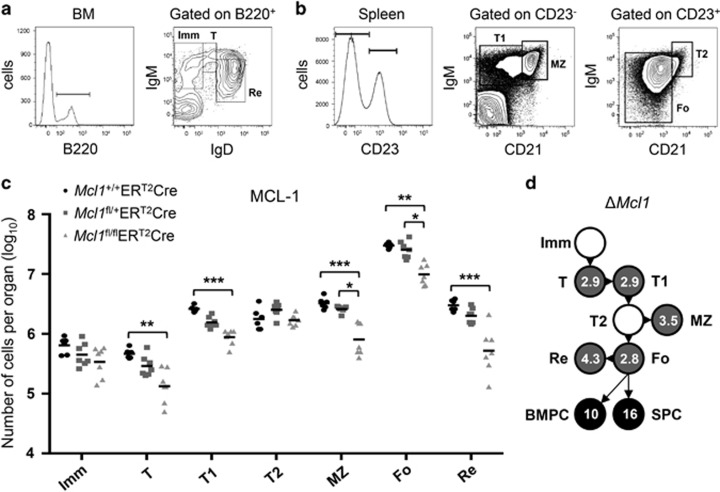
B-lymphoid cells are reliant on MCL-1 expression throughout development. (**a** and **b**) Flow cytometric analysis of cells from the BM and spleen of a control (*Mcl1*^+/+^ER^T2^Cre) mixed-BM chimeric mouse. (**a**) Subset representation in the BM. Abbreviations adjacent to outlined areas indicate the corresponding B-cell population with immature (Imm; B220^+^IgM^+^IgD^−^), transitional (T; B220^+^IgM^hi^IgD^int^) or recirculating mature (Re; B220^+^IgM^int^IgD^hi^) B cells in BM. (**b**) Subset representation in the spleen. Abbreviations adjacent to outlined areas indicate the corresponding B-cell population with transitional type 1 (T1; CD23^−^IgM^+^CD21^lo^), transitional type 2 (T2; CD23^+^IgM^hi^CD21^hi^), marginal zone (MZ; CD23^−^IgM^hi^CD21^hi^) or mature follicular (Fo; CD23^+^IgM^+^CD21^+^) B cells. (**c**) Absolute numbers of B-cell populations from the BM and spleen of mixed-BM chimeras after induced deletion of one (*fl/+*) or two (*fl/fl*) alleles of *Mcl1*. *In vivo* Cre-mediated gene deletion was induced by two tamoxifen treatments on consecutive days by oral gavage, with mice sacrificed 48 h after the first dose. Cell numbers were calculated based on total cell numbers per organ and gates of dot plots from flow cytometric analysis as shown in (**a**) or (**b**). Efficiency of *Mcl1* gene deletion was shown previously.^15^ Data represent two independent experiments and six or seven animals per group. (**d**) Fold reduction of B-cell numbers throughout development comparing control *Mcl1*^+/+^ER^T2^Cre to *Mcl1*^fl/fl^ER^T2^Cre mixed-BM chimeras, based on numbers shown in (**c**) and on previously published data.^16^ *P⩽0.05, **P⩽0.01, ***P⩽0.001 (statistical significance was calculated between the groups using a Kruskal–Wallis test followed by a *post hoc* Dunn's multiple comparisons test)

The importance of MCL-1 for B-lymphocyte development in mice has been demonstrated by others using CD19-Cre-mediated deletion of *Mcl1*^*fl*^ alleles. These studies revealed a significant reduction in all B-cell subsets from the early pro-B-cell stage onwards,^20^ this being the stage of Cre-recombinase expression and thus deletion of *Mcl1*^*fl*^. These experiments, however, could not reveal a role for MCL-1 beyond the pro-B-cell stage as differentiation was halted at this point. To determine if there is differentiation stage-specific sensitivity to *Mcl1* deletion in the B-cell lineage specifically, we used a mouse model in which *Mcl1*^*fl*^ could be efficiently deleted in established B-cell populations of adult mice by delivery of tamoxifen to activate a conditional Cre^ERT2^ recombinase.^16^ Deletion of both *Mcl1*^*fl*^ alleles in this way rapidly reduced the absolute number of cells in transitional and mature B-cell subsets in the spleen and bone marrow (BM) (Figures 1a–d). The impact of *Mcl1*^*fl*^ deletion in PC in this setting has been previously reported^16^ and the fold difference in PC number after tamoxifen-mediated *Mcl1*^*fl*^ deletion is included (Figure 1d).

**Figure 2:**
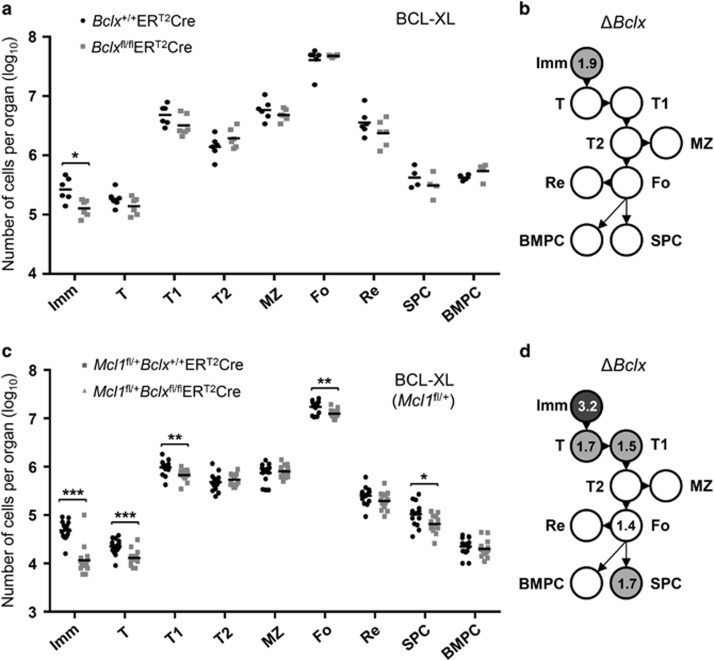
Reduced expression of MCL-1 sensitizes B cells to deletion of *Bclx*. (**a**) Absolute numbers of cells from the spleen and BM of mixed-BM chimeras after induced deletion of both (*fl/fl*) alleles of *Bclx* calculated as in Figure 1. *In vivo* Cre-mediated gene deletion was induced by two tamoxifen treatments on consecutive days by oral gavage, with mice killed 4 days after the first dose. This longer interval allowed for the loss of BCL-XL protein, which has a longer half-life (~24 h) than MCL-1 (<1 h).^15^ Efficiency of *Bclx* gene deletion was shown previously.^16^ Data represent two independent experiments and six animals per group. (**b**) Fold reduction of B-cell numbers throughout development comparing control *Bclx*^+/+^ER^T2^Cre and *Bclx*^fl/fl^ER^T2^Cre mixed-BM chimaeras, based on numbers shown in (**a**). (**c**) Absolute numbers of cells after induced deletion of one (*fl/+*) allele of *Mcl1* and both (*fl/fl*) alleles of *Bclx*. Mice were killed 4 days after the first tamoxifen treatment. Data represent three independent experiments and 10–15 animals per group. (**d**) Fold reduction of B-cell numbers comparing *Mcl1*^fl/+^*Bclx*^+/+^ER^T2^Cre and *Mcl1*^fl/+^*Bclx*^fl/fl^ER^T2^Cre mixed-BM chimeras, based on numbers shown in (**c**). *P⩽0.05, **P⩽0.01, ***P⩽0.001 (statistical significance was calculated between the groups using a Mann–Whitney test)

To determine the contribution of BCL-XL to the sustained survival of B-cell subsets, we created BM chimeric mice in which *Bclx*^*fl*^ alleles could be inducibly deleted in the B-cell lineage.^16^ Loss of BCL-XL significantly reduced only the BM immature B-cell population (Figures 2a and b). Because of the relatively modest impact of *Bclx*^*fl*^ deletion, we next examined the consequences of combined inducible deletion of both alleles of *Bclx*^*fl*^ plus one allele of *Mcl1*^*fl*^. In addition to a reduction in the numbers of immature B cells caused by loss of BCL-XL expression, additional deletion of one *Mcl1*^*fl*^ allele also significantly reduced the numbers of transitional B cells in the BM as well as T1, follicular B and PC in the spleen (Figures 2c and d). The fold differences shown are calculated comparing *Mcl1*^*fl/+*^*Bclx*^*fl/fl*^ER^T2^Cre with *Mcl1*^*fl/+*^*Bclx*^*+l+*^ER^T2^Cre, thus revealing the impact of *Bclx* deletion in a setting in which MCL-1 expression is reduced with 50% (Figure 2d).

**Figure 3:**
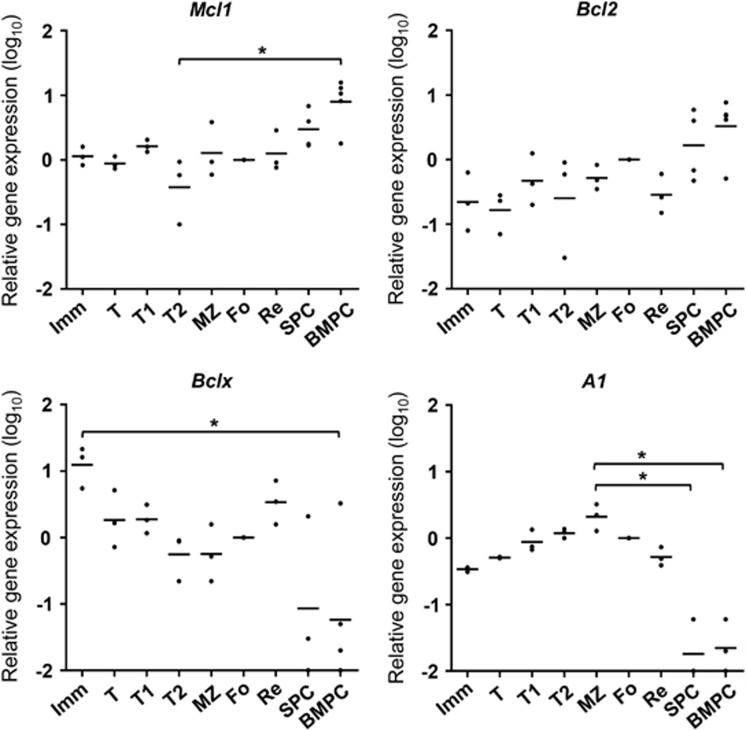
Gene expression of *Mcl1*, *Bcl2*, *Bclx (Bcl2l1)* and *A1 (Bcl2a1)* throughout B-cell development. Quantitative PCR analysis of mRNA encoding pro-survival members of the BCL-2 family in different B-cell populations. FACS-sorting was performed as shown in Figures 1a and b. Data are normalized to expression of the housekeeping gene *Hprt* and presented relative to expression in Fo B cells, set as 0 (log_10_ of 1). Numbers in the graph indicate relative expression and represent three to five independent experiments, each performed in duplicate. *P⩽0.05 (statistical significance was calculated between the groups using a Kruskal–Wallis test followed by a *post hoc* Dunn's multiple comparisons test)

As BCL-XL is mainly important for cell survival during early B-cell development, *Bclx* gene expression was measured and related to gene expression of *Mcl1*, *Bcl2* and *A1*. This analysis revealed that, contrary to expression of *Mcl1* and *Bcl2*, *Bclx* transcription peaks early during B-cell development and is highest in immature B cells (Figure 3). Expression of both *Mcl1* and *Bcl2* peaks in terminally differentiated PC, whereas expression of *A1* is relatively low in immature B cells and PC but high in T1 and T2 transitional cells and mature marginal zone and follicular B cells (Figure 3).

**Figure 4:**
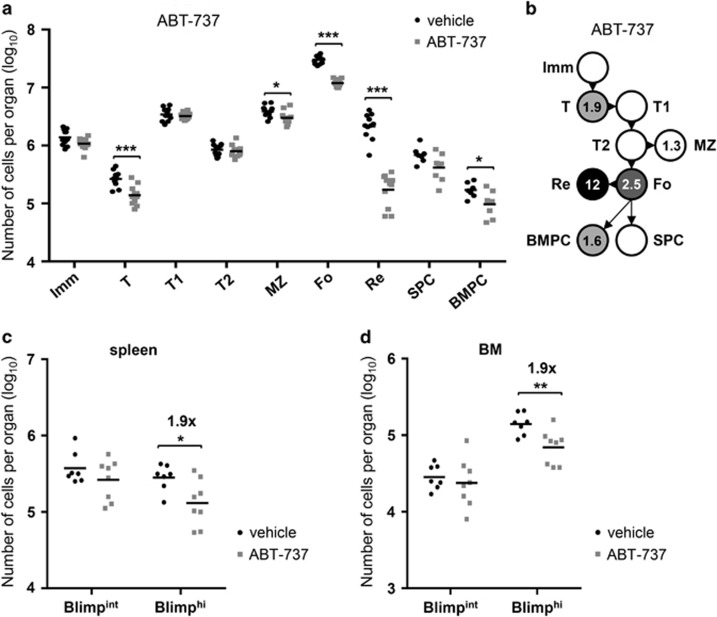
Treatment with BH3-mimetic ABT-737 reduces Blimp-1^hi^ plasma cells. (**a**) Absolute numbers of cells after treatment of wild-type mice with 75 mg/kg ABT-737 for 5 days, calculated as in Figure 1. Data represent three experiments and 7–11 animals per group. (**b**) Fold reduction of B-cell numbers comparing wild-type+vehicle and wild-type+ABT-737, based on numbers shown in (**a**). (**c–d**) Absolute numbers of Blimp-1^int^ and Blimp-1^hi^ PC after treatment of Blimp-1-GFP reporter mice (*Prdm1*^GFP^)^21^ with ABT-737 as in (**a**). Shown are PC in spleen (**c**) and BM (**d**). Data in (**c**) and (**d**) represent two experiments and 7–8 animals per group. *P⩽0.05, **P⩽0.01, ***P⩽0.001 (statistical significance was calculated between the groups using a Mann–Whitney test)

ABT-737 potently binds to BCL-2, BCL-XL and BCL-W in Biacore assays, but has a clear preference for blocking BCL-2 in living cells in culture and within the whole mouse.^17, 18^ Thus, its effects on cell survival *in vivo* can mainly be attributed to inhibition of BCL-2. ABT-737 was chosen for reasons of cost and availability, and when administered by intraperitoneal injection, becomes available to all relevant organs.^19^ ABT-737 has a very similar binding specificity and affinity as the BH3-mimetic ABT-263/Navitoclax that has improved bioavailability.^21^ Treatment of mice with ABT-737 promotes apoptosis of mature follicular B cells in the spleen and recirculating B cells in the BM,^19, 22^ it also affects the survival of newly generated PC *en route* to the BM, but does not markedly affect preexisting PCs.^19^ We examined the impact of ABT-737 on PC in more detail and found that the total PC population in the BM, but not the spleen, was mildly, but significantly, reduced upon treatment (Figures 4a and b). In addition, we found that mature follicular and recirculating B cells were strongly reduced after treatment, as expected from previous studies. We also observed a significant reduction in the numbers of marginal zone B cells and transitional B cells in the BM (Figures 4a and b). Next, we compared short-lived (Blimp-1^int^) with long-lived (Blimp-1^hi^) PC for sensitivity to ABT-737 by treating Blimp-1-GFP reporter mice (in which GFP is expressed from the gene encoding Blimp-1 (*Prdm1*^GFP^))^23^ with this drug. Interestingly, we observed that in both the spleen and BM, only the Blimp-1^hi^ PC were affected by ABT-737 treatment (Figures 4c and d). As, in contrast to the spleen, the majority of PC in the BM are long-lived Blimp-1^hi^ PC,^23^ these observations are in line with the results on the total PC population in Figure 4a.

**Figure 5:**
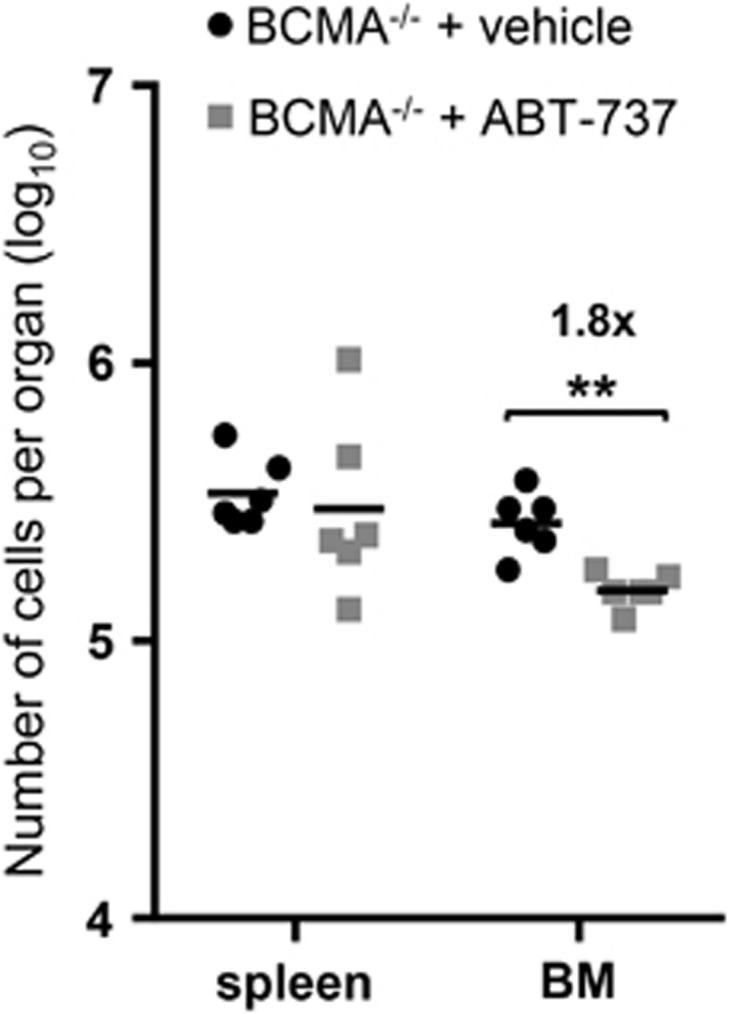
Plasma cells that persist in the absence of TNF receptor BCMA remain sensitive to ABT-737. Absolute numbers of PC (CD138^+^B220^lo^) after ABT-737 treatment of lethally irradiated mice reconstituted with *Bcma*^−/−^ BM, performed as in Figure 4. Data represent one experiment and six animals per group. **P⩽0.01 (Statistical significance was calculated between the groups using a Mann–Whitney test)

**Figure 6:**
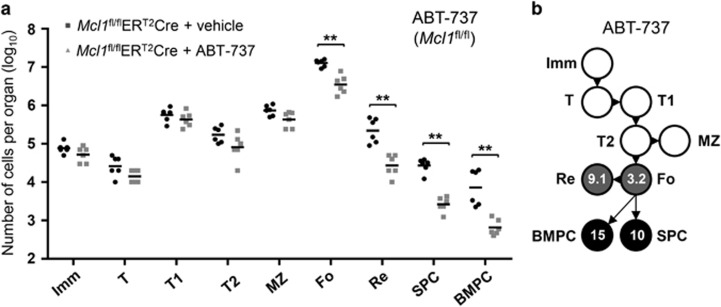
Reduced expression of MCL-1 sensitizes plasma cells to BCL-2 inhibition with ABT-737. (**a**) Absolute numbers of cells after induced deletion of both (*fl/fl*) alleles of *Mcl1* and treatment with ABT-737. Mice were killed 2 days after the first treatment with tamoxifen (treated for 2 consecutive days) and 5 days after treatment with 75 mg/kg ABT-737 (treated for 5 consecutive days). Data represent two experiments and six animals per group. (**b**) Fold reduction of B-cell numbers comparing *Mcl1*^fl/fl^ER^T2^Cre+vehicle and *Mcl1*^fl/fl^ER^T2^Cre+ABT-737, based on numbers shown in (**a**). **P⩽0.01 (statistical significance was calculated between the groups using a Mann–Whitney test)

We have shown previously that MCL-1 expression is essential for PC survival and that signaling through the TNF receptor (TNFR) family member BCMA promotes *Mcl1* transcription in BM PC.^16^ In fact, we have shown that *Mcl1* gene expression was 3.4-fold lower in *Bcma*^−/−^ BM PC as compared with wild-type BM PC.^16^ Mice deficient for BCMA in haematopoietic cells were treated with ABT-737 as before and the impact on PC maintenance was measured. Although PC numbers in the BM were reduced in *Bcma*^−/−^ mice,^16, 24^ those PC that survive in the absence of BCMA-signaling remained as sensitive to treatment with ABT-737 as do those BM PC that survive in its presence (Figure 5). These results suggest the existence of non-overlapping roles between MCL-1, albeit reduced in BCMA-deficient BM PC, and BCL-2, the main target of ABT-737 in mice.^17, 18^ Therefore, we further tested the combinatorial dependence of MCL-1 and BCL-2 expression on PC survival. The combined impact of ABT-737 treatment with deletion of both *Mcl1*^*fl*^alleles was examined. Treatment with ABT-737 in the absence of MCL-1 revealed a similar impact on follicular and recirculating B cells as compared with ABT-737 treatment in wild-type mice (Figures 6a and b). However, ABT-737 treatment in the absence of MCL-1 greatly augmented the reduction in PC populations in both the spleen and BM owing to MCL-1 (Figures 6a and b).

**Figure 7:**
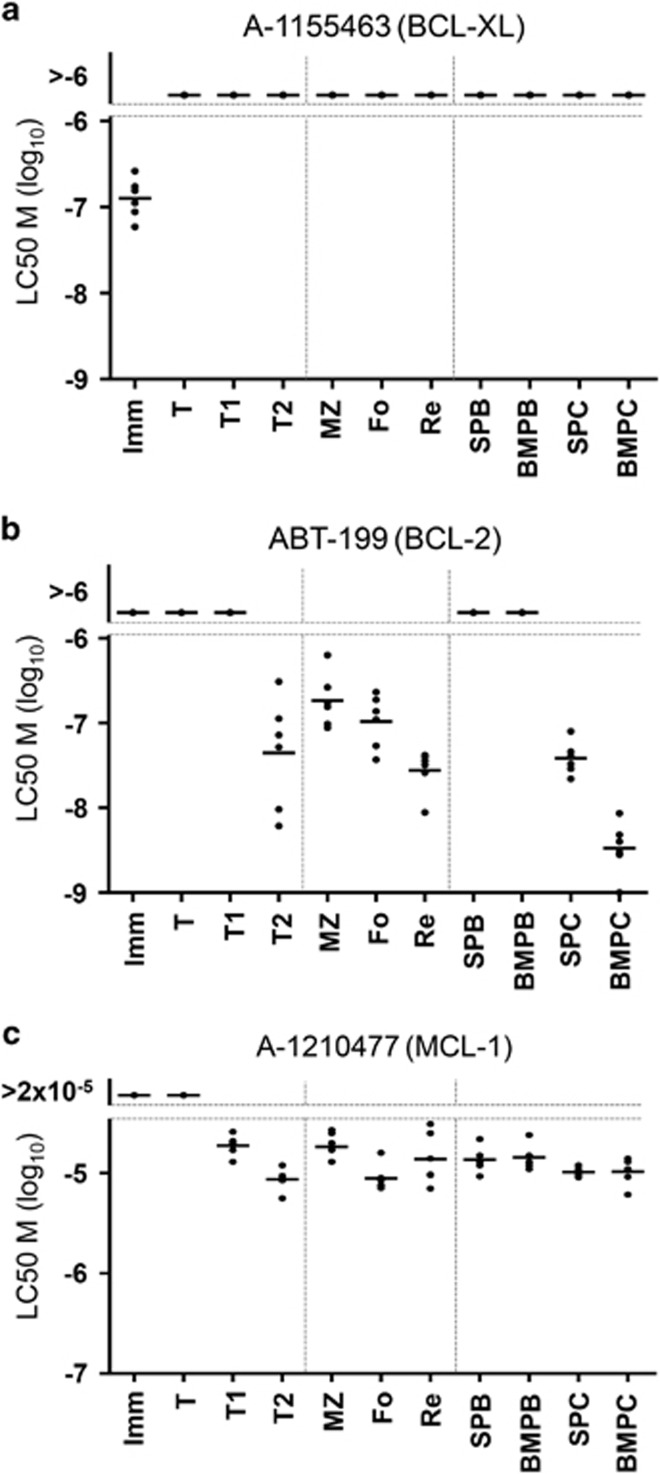
Sensitivity of B cells to highly specific BH3-mimetics that target BCL-XL, BCL-2 or MCL-1. Response of diverse freshly isolated mouse B-cell subsets to (**a**) the BCL-XL-selective BH3-mimetic A-1155463, (**b**) the BCL-2-selective BH3-mimetic ABT-199 or (**c**) the MCL-1-selective BH3-mimetic A-1210477. Data represent cells from the spleen or bone marrow of six individual mice

To complement our *in vivo* studies, we examined dependence on BCL-2, BCL-XL or MCL-1 expression using novel and highly specific BH3-mimetics with murine splenocytes and BM cells in culture. Identical to *Bclx* gene deletion experiments *in vivo* (Figures 2a and b), treatment with the BCL-XL-specific inhibitor, A-1155463,^25^ only reduced the viability of immature B cells (Figure 7a). Sensitivity to the BCL-2-specific inhibitor ABT-199/Venetoclax^13^ was comparable to our observations with *in vivo* experiments using ABT-737 (Figures 4a and b), promoting apoptosis of mature marginal zone, follicular and recirculating B cells. In addition, we observed impaired survival of transitional type 2 cells from the spleen (Figure 7b). The latter was not observed in our *in vivo* experiments with ABT-737, but was previously described in an identical experimental set-up using ABT-199.^26^ As expected from our experiments in Figures 4C and D, ABT-199 promoted apoptosis of fully differentiated PC, whereas early plasmablasts (PB, comparable to Blimp-intermediate PC) were unaffected by treatment with this drug (Figure 7b). Next we tested the role of MCL-1 in survival of B-cell subsets using the MCL-1 inhibitor A-1210477. Although A-1210477 is highly specific for MCL-1, it lacks potency to reveal subtle differences in MCL-1 dependence.^27^ In line with our gene deletion experiments presented in Figure 1 and previous findings,^16^ we observed that the transitional as well as mature B-cell- and PC-subsets, but not immature B cells, were sensitive to treatment with this drug (Figure 7c). In contrast to our *in vivo* experiments, we found that transitional type 2 cells in the spleen were sensitive, but transitional B cells from the BM were insensitive to A-1210477. The differential dependence on BCL-2 or MCL-1 expression in transitional B cells from the BM or transitional type 2 B cells from the spleen between our *in vitro* or *in vivo* models may be possible owing to the potency of the BH3-mimetics or the role of the protective microenvironment *in vivo*.

## Discussion

Selective targeting of pro-survival BCL-2 family members can provide an attractive strategy for treating B-cell malignancies, either as single treatment or in combination with additional treatment modalities. This is exemplified by the recent discovery and use of the BCL-2-specific inhibitor ABT-199 (Venetoclax).^13^ Venetoclax has proven successful in multiple clinical trials and is a promising new treatment for CLL, certain non-Hodgkin lymphomas and other malignancies.^14, 28^ Its predecessor, Navitoclax/ABT-263 (the orally bioavailable form of ABT-737), targets not only BCL-2, but also BCL-XL and BCL-W. As expression of BCL-XL is required for maintenance of platelets, Navitoclax evoked severe thrombocytopenia in clinical trials as an unwanted side-effect.^28^ The main target of ABT-737 in mice, however, was shown to be BCL-2 and not BCL-XL or BCL-W.^17, 18^ In addition to BCL-2 inhibition, novel drugs have been described that more specifically inhibit only BCL-XL (WEHI-539 and A-1155463)^25, 29^ or MCL-1 (A-1210477).^27^ As various tumor types depend on MCL-1 or BCL-XL expression,^14^ it is only a matter of time before specific inhibitors targeting these molecules will also find their way into the clinic. However, the importance of MCL-1, BCL-2 and BCL-XL expression for the survival of healthy cell subsets *in vivo* is still incompletely understood. Thus, treatment with these specific inhibitors used alone or in combination may elicit unwanted side-effects, as seen with the impact of BCL-XL inhibition on platelets. A thorough understanding of the effects of BH3-mimetic drugs on normal cells should aid the development of a tailored treatment design that minimizes side-effects. Conversely, knowing which cell subsets are sensitive to the different inhibitors may provide clues on how to treat their malignant counterparts.

To assess the individual and overlapping roles of MCL-1, BCL-2 and BCL-XL in the maintenance of different B-cell subsets, we examined mice where the *Mcl1* and/or *Bclx* genes were inducibly deleted in the B-cell lineage. This was performed in combination with ABT-737 treatment. Our findings demonstrate a reliance on MCL-1 expression throughout the B-cell lineage and indicate that even short-term loss of MCL-1 expression – for <2 days – will deplete B-cell subsets at multiple stages of development (Figures 1 and 7c). In contrast, loss of BCL-XL expression, even up to 4 days, only affects the maintenance of immature B cells. The specific dependence of this B-cell subset on expression of BCL-XL was confirmed using the BCL-XL-specific inhibitor, A-1155463 (Figure 7a). Hemizygous deletion of *Mcl1* did, however, sensitize transitional B cells and splenic PC to loss of BCL-XL (Figure 2). Previous reports have shown that treatment of mice with ABT-737 results in a decrease of follicular and recirculating mature B cells.^19, 22^ It inhibited the induction of newly generated PC, but left preexisting PC intact.^19^ We show here that also long-lived terminally differentiated (Blimp-1^hi^) PC, but not short-lived Blimp-1^int^ PC, are reduced after treatment with ABT-737 (Figure 4). In the above-mentioned experiments ABT-737 was chosen to study the role of BCL-2, its main *in vivo* target, for reasons of cost and availability. We did, however, also use the BCL-2-specific BH3-mimetic ABT-199 on isolated BM and splenic B cells in culture. These experiments confirmed to a large extend our *in vivo* findings when using ABT-737, with mature marginal zone, follicular and recirculating B cells sensitive to BCL-2 inhibition (Figure 7b). We also confirmed our *in vivo* findings, proving that long-lived (B220^low^ or Blimp-1^hi^) PC depend on BCL-2 expression, whereas recently generated, or short-lived (B220^hi^ or Blimp-1^int^) PC do not (Figure 7b). The notion that mainly Blimp-1^hi^ PC are sensitive to this treatment correlates with previous findings showing that *Bcl2* expression is higher in these cells compared to Blimp-1^int^ PC.^16^ As PC seem to be affected both by deletion of *Mcl1* and treatment with ABT-737, the combined impact on cell survival was examined. BCMA, a TNFR family member expressed specifically on PC, promotes survival of BM PC via transcriptional induction of *Mcl1* and, as a consequence, the number of BM PC in *Bcma*^−/−^ mice is significantly reduced.^16, 24^ ABT-737 treatment of mice reconstituted with *Bcma*^−/−^ BM showed a similar fold reduction in the numbers of BM PC as seen in wild-type mice treated with ABT-737 (Figures 4 and 5). This means that reduced MCL-1 expression, caused by the absence of BCMA-signaling, and inhibition of BCL-2 with ABT-737 have additive effects on the reduction of BM PC. The combined effects of BCL-2 inhibition and reduced MCL-1 expression were further examined by treating mice with ABT-737 together with genetically deleting one or both alleles of *Mcl1*. These experiments revealed that B-cell populations in mice with hemizygous deletion of *Mcl1* are equally sensitive to treatment with ABT-737 compared with wild-type mice (data not shown). However, PC in mice with homozygous deletion of *Mcl1* are strikingly sensitive to ABT-737 (Figure 6). These synergistic effects between inhibited BCL-2 and loss of MCL-1 expression seem to be specific for PC and opens up a potential therapeutic strategy for deletion of auto-reactive or malignant PC in patients. This finding does, however, argue that the reduction in MCL-1 expression needs to be substantial (more than 3.4-fold based on experiments with *Bcma*^*−/−*^ PC) to observe synergism with ABT-737 (BCL-2 inhibition). As the number of PC is already strongly decreased in the absence of MCL-1,^16^ the additional impact of ABT-737 results in a >100-fold decrease in the number of PC in both the spleen and BM (Figure 6a).

**Figure 8:**
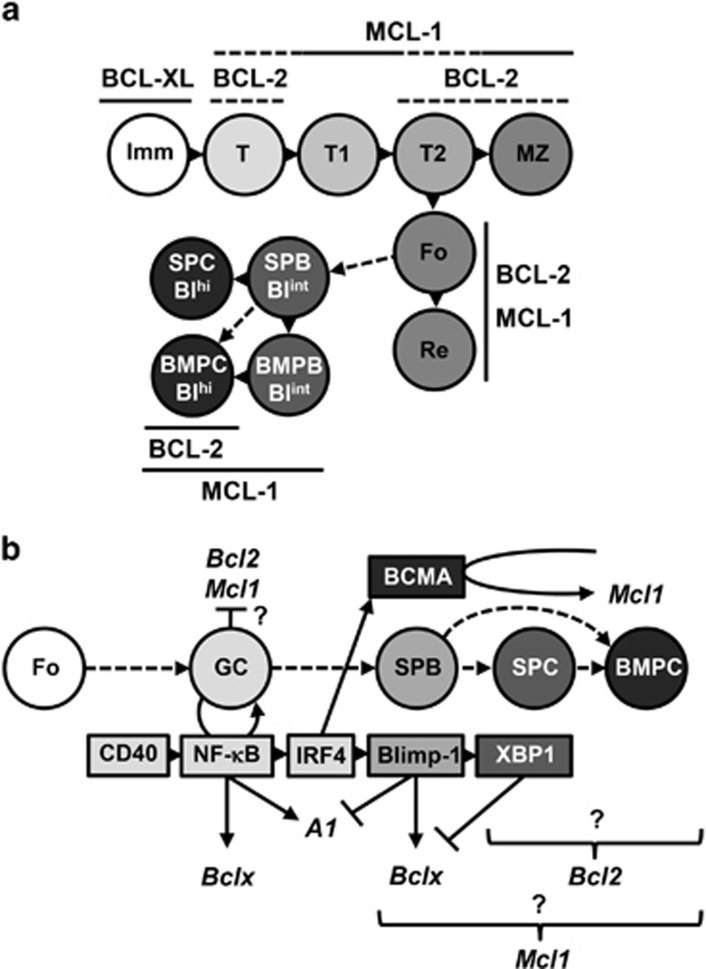
Differential dependence on – and expression of – pro-survival BCL-2 family members throughout B-cell development. (**a**) Dependence on expression of MCL-1, BCL-2 and BCL-XL throughout B-cell development. Solid lines indicate direct reliance on indicated BCL-2 family protein based on both *in vivo* data and *ex vivo* experiments using BH3-mimetics, whereas dotted lines indicate reliance on either *in vivo* data or *ex vivo* experiments with BH3-mimetics. Data are a summary of Figures 1c,2a,4a, 4c, 4d,6a and 7. (**b**) Regulation of *Mcl1, Bcl2, Bclx (Bcl2l1)* and *A1 (Bcl2a1)* gene expression in germinal center (GC) B cells and PC. BCL-6-mediated inhibition of PC master regulator Blimp-1 can be abrogated by activation of IRF4.^33^ This can be achieved by NF-*κ*B activation following CD40 ligation.^34^ Activated NF-κB can promote transcription of pro-survival BCL-2 family protein A1 (BFL-1),^35^ which is subsequently inhibited by Blimp-1 when cells differentiate to PC.^16^ Blimp-1 can promote transcription of *Bclx,* which is subsequently repressed by XBP-1.^30, 31^ Expression of *Mcl1* is transcriptionally induced by stimulation of BCMA in the BM microenvironment,^16^ however, the mechanism of increased *Mcl1* transcription in splenic PC remains unclear. Repression of *Bcl2* has been observed in the early GC but is subsequently re-expressed in mature PC,^16^ although the upstream mediators are currently unknown

Collectively, these studies reveal the differential requirement and expression of specific anti-apoptotic BCL-2 family proteins during different stages of B-cell development (Figure 8a). It also shows that reduced expression of MCL-1 sensitizes multiple B-cell subsets to inhibition of BCL-XL or BCL-2. Recent publications shed light on the mechanism of the transient BCL-XL expression in newly generated PC, that has been described previously.^16^ Expression of Blimp-1 represses A1, but has also been shown to bind to the *BCLX* gene promoter and promote its transcription.^30, 31^ Activation of XBP-1, downstream of Blimp-1, subsequently represses *BCLX* (Figure 8b).^31^ The transient expression of BCL-XL is thought to safeguard PC *en route* to protective BM niches,^15, 20^ and we show here that BCL-XL does contribute significantly to the survival of splenic PC when MCL-1 expression is limited. The mechanism(s) that promote(s) *BCL2* transcription in Blimp-1hi PC^16^ is currently unresolved. Our results suggest that combining inhibition of BCL-2 with drugs that reduce the expression or activity of MCL-1 may prove valuable for targeting auto-reactive or malignant PC in autoimmune disease or multiple myeloma, respectively.

## Materials and Methods

### Mice

C57BL/6 mice, *Prdm1*^GFP/+^ mice,^23^ *Mcl1*^flox^ mice,^16^ *Bclx*^flox^ mice,^16^ Rosa26-Cre^ERT2^ (hereafter, called Cre^ERT2^) mice^16^ (Taconic Artemis, Hudson, NY, USA) and *μ*MT-deficient mice^32^ were bred and maintained at the animal facilities of the Walter and Eliza Hall Institute (WEHI). All gene-targeted mice were maintained on a C57BL/6 background. *Bcma*^−/−^ mice^16^ were on a C57BL/6-and-C3H/He mixed genetic background. All animal procedures were approved by the WEHI Animal Ethics Committee. BM-reconstituted mice were generated as described.^16^ In brief, lethally irradiated C57BL/6 (Ly5.1^+^) mice were reconstituted with 80% B-cell-deficient (*μ*MT) BM plus 20% of either (Ly5.2^+^) Cre^ERT2^, *Mcl1*^fl/+^Cre^ERT2^, *Mcl1*^fl/fl^Cre^ERT2^, *Bclx*^fl/fl^Cre^ERT2^ or *Mcl1*^fl/+^*Bclx*^fl/fl^Cre^ERT2^ BM. Activation of the Cre^ERT2^ conditional recombinase to achieve deletion of *lox*P-flanked *Mcl1* or *Bclx* alleles was performed by oral gavage of tamoxifen on 2 successive days, as described.^16^ Alternatively, mice deficient for BCMA were generated by reconstitution of lethally irradiated C57BL/6 (Ly5.1^+^) mice with 100% *Bcma*^−/−^ BM (Ly5.2^+^). ABT-737 (Abbott Laboratories, Abbott Park, IL, USA) or vehicle control were prepared and administered at 75 mg/kg body weight for 5 consecutive days as described.^19^

### Flow cytometry

Single-cell suspensions were stained with the following fluorochrome-conjugated monoclonal antibodies: anti-B220 (RA3-6B2), anti-IgM (331.12), anti-IgD (11.26C), anti-CD23 (B3B4), anti-CD21 (7E9), anti-Fc*γ*R (2.4G2), anti-human CD4 (OKT4) all produced in-house, anti-CD138 (281.2; BD Biosciences, San Jose, CA, USA) and anti-CD45.2 (104; BD Biosciences). Stained cells were analyzed on a FACSCanto II cytometer (BD Biosciences). B-cell populations were sorted from spleen and BM using a MoFlo cytometer (DAKO Cytomation Ltd, Ely, UK) to a purity of 98%.

### Quantitative PCR

RNA isolation, quantitative real time PCR and primer sequences are as described.^15, 16^

### BH3-mimetics and apoptosis assays

Single-cell suspensions from the spleen or BM were cultured with A-1210477 (catalog number CT-A121, ChemieTek, Indianapolis, IN, USA) at a concentration range of 0, 1, 5 or 20 μM, A-1155463 (catalog number CT-A115, ChemieTek) at 0, 10, 100 or 100 nM, or ABT-199 (catalog number A0776, LKT Laboratories, St Paul, MN, USA) at 0, 10, 100 or 1000 nM for 20 h at 37ºC. Cell viability after treatment was assessed by flow cytometry using the TO-PRO-3 dye (catalog number T3605, ThermoFisher, Waltham, MA, USA) after gating on specific B-cell subsets using the gating strategy as shown in Figures 1a and b or as previously published for PC.^16^ Specific apoptosis was calculated by measuring the altered percentage of TOPRO3^−^ (live) cells within indicated B-cell populations, compared with untreated cells. LC50 values were subsequently calculated using Excel and Graphpad Prism software, and specific apoptosis values after incubation with a concentration range of the inhibitors.

### Statistical analysis

Statistical significance was determined using a Mann–Whitney test when comparing two groups of mice (Figures 2a, c,4a, c, d,5 and 6a), or a Kruskal–Wallis test followed by a *post hoc*Dunn's multiple comparisons test in case multiple groups of mice or cell subsets were compared (Figures 1c and 3).

## Abbreviations

BCL-2: B-cell lymphoma 2
BCL2A1 (A1): B-cell lymphoma 2-related protein A1
Bcl2l1: B-cell lymphoma 2 like 1
BCL-6: B-cell lymphoma 6
BCL-W: B-cell lymphoma W
BCL-XL: B-cell lymphoma extra-large
BCMA: B-cell maturation antigen
BFL-1: Bcl-2-related gene in fetal liver 1
BH3: Bcl-2 homology domain 3
Blimp-1: B-lymphocyte-induced maturation protein 1
BM: bone marrow
CLL: chronic lymphocytic leukemia
ERT2: estrogen receptor type 2
Fl: floxed
Fo: follicular
GC: germinal center
GFP: green fluorescent protein
Hprt: hypoxanthine-guanine phosophoribosyltransferase
Imm: immature
IRF4: interferon regulatory factor 4
LC50: lethal concentration 50
MCL-1: myeloid cell leukemia 1
mRNA: messenger ribonucleic acid
MZ: marginal zone
NF-*κ*B: nuclear factor kappa-light-chain-enhancer of activated B cells
PB: plasmablast
PC: plasma cell
PCR: polymerase chain reaction
Prdm1: PR domain zinc finger protein 1
Re: recirculating
RNA: ribonucleic acid
Rosa26: gene locus used for constitutive ubiquitous gene expression in mice
T1: transitional type 1
T2: transitional type 2
TNFR: tumor necrosis factor receptor
WEHI: Walter and Eliza Hall institute
XBP-1: X-box binding protein 1
*μ*MT: IgM transmembrane tail exons
